# Serum metabolome alterations in patients with early nonalcoholic fatty liver disease

**DOI:** 10.1042/BSR20220319

**Published:** 2022-10-18

**Authors:** Xuemei Wang, Benchen Rao, Haiyu Wang, Chao Liu, Zhigang Ren, Zujiang Yu

**Affiliations:** 1 Department of Infectious Diseases, the First Affiliated Hospital of Zhengzhou University, Zhengzhou 450052, China; 2 Gene Hospital of Henan Province; Precision Medicine Center, the First Affiliated Hospital of Zhengzhou University, Zhengzhou 450052, China; 3 Shanghai Mobio Biomedical Technology Co., Ltd., Shanghai 201111, China

**Keywords:** Diagnostic Markers, Diagnostic Model, Early-stage Nonalcoholic Fatty Liver, Metabolites, Serum Metabolomics

## Abstract

**Background:** Although metabolomic analysis for patients with nonalcoholic fatty liver disease (NAFLD) was a promising approach to identify novel biomarkers as targets for the diagnosis of NAFLD, the serum metabolomics profile of early-stage NAFLD patients from central China remain unknown. **Objective:** The aim of the present study was to explore the metabolic characteristics of patients with early-stage NAFLD based on the ultra-performance liquid chromatography-tandem mass spectrometry (UPLC-MS/MS) technology, to identify differential metabolites and perform functional analysis, and especially, to establish a novel early NAFLD clinical diagnostic tool. Results: Compared with healthy controls, serum metabolite species increased significantly in early stage NAFLD patients. Expression of 88 metabolites including 1-naphthylmethanol, rosavin, and theophylline were up-regulated in early NAFLD, while 68 metabolites including 2-hydroxyphenylacetic acid and lysophosphatidylcholine (24:1(15Z)) were down-regulated. The early NAFLD classifier achieved a strong diagnostic efficiency in the discovery phases (80.99%) and was successfully verified in the validation phases (75.23%). **Conclusions:** These results advance our understanding about the composition and biological functions of serum metabolites of early NAFLD. In addition, serum metabolic markers can serve as an efficient diagnostic tool for the early-stage NAFLD.

## Introduction

Nonalcoholic fatty liver disease (NAFLD) is a metabolic associated fatty liver disease, including early nonalcoholic fatty liver (NAFL) and nonalcoholic steatohepatitis (NASH), and the later can further progress to cirrhosis and even hepatocellular carcinoma (HCC) [1,2]. NAFLD is strongly associated with the high prevalence of metabolic diseases, including diabetes, dyslipidemia, and obesity [3,4]. As the most common chronic liver disease worldwide, although the global prevalence of NAFLD has risen dramatically to 25.24% of the global adult population, there are no approved drug treatments for NAFLD [5]. Although the conventional detection and definitive diagnosis approaches for NAFLD are hepatic ultrasonography and biopsy, neither approach can further investigate the underlying mechanisms regarding the occurrence and progression of NAFLD [6]. Moreover, limitations including invasive and expensive prevent liver biopsy from being a widely used screening method or diagnostic tool for early NAFLD [7]. Hence, a novel, noninvasive and simple medical method based on serum biomarkers, which can provide a molecular mechanism for the diagnosis of early NAFLD, is urgently needed.

Metabolomics can directly detect the physiological and pathological status of the individuals, providing the comprehensive and direct characterization for researchers [8]. Furthermore, metabolomics has attracted much attention for its powerful diagnostic, disease severity classification and result prediction potential in various diseases, such as HCC [9] and chronic pancreatitis [10]. Liver is known to be an important digestive organ involved in multiple biochemical reactions and metabolic processes. The composition of human serum metabolites is influenced by a variety of factors, such as disease state, intestinal flora disorders, ethnicity, and region [11]. Metabolomics study showed that the plasma levels of glycocholate, taurocholate, and glycochenodeoxycholate were significantly elevated in NAFLD patients [6,12]. In addition, Dong et al. performed clinical study through a urinary mass spectrometry-based metabolomics and found compared with the healthy individuals, increased levels of valine, arginine, and citrulline in patients with NAFLD [13]. Notably, they further found only pyroglutamic acid could distinguish between NAFLD and NASH [13]. Recently, study identified 55 metabolites in plasma that differed significantly between ultrasound diagnosed NAFLD patients and the healthy population [14]. Additionally, the investigators further identified 15 serum biomarkers that can distinguish patients with NAFLD and the healthy individuals through receiver operating characteristic curve (ROC) analysis [14]. Collectively, these studies indicated that metabolomic analysis for patients with early NAFLD is a promising approach to identify novel biomarkers as the diagnosis targets for NAFLD [15].

To our knowledge, the serum metabolomics profile of patients with early NAFLD in central China remains unknown. Our present study mainly focused on the early stage NAFLD, including NAFL and NASH, considering the unique metabolomic profile for cirrhosis [16]. Therefore, the present study aimed to report the serum metabolomic profiles of early NAFLD patients diagnosed by ultrasound and healthy volunteers from central China using the ultra-performance liquid chromatography-tandem mass spectrometry (UPLC-MS/MS), and to establish a diagnostic model that could distinguish the early-stage NAFLD patients from healthy individuals based on the metabolic markers. Importantly, through mining the biological functions of differential metabolites through pathway analysis, the present study will complement the mechanisms of NAFLD progression and provide a molecular research basis for NAFLD diagnosis and therapeutic targets.

## Materials and methods

### Participants inclusion and exclusion

The present study was conducted following the principles of prospective specimen collection, retrospective analysis with blind evaluation and the ethical guidelines of the 1975 Declaration of Helsinki [17]. It was approved by the Ethics Committee of the First Affiliated Hospital of Zhengzhou University (2021-KY-0715-002). Written informed consent was signed by each participant (Supplementary Data).

All participants were obtained from the outpatient clinic of the First Affiliated Hospital of Zhengzhou University, China, from July 2020 to January 2021. We performed a rigorous questionnaire for the participants, including age, height, weight, drinking history, and medical history. According to the clinical guideline of NAFLD updated in 2018 [18], the diagnosis of early NAFLD was based on hepatic steatosis shown by hepatic ultrasonography or biopsy. The ultrasound images characteristics: diffusely enhanced liver echogenicity, liver vascular blurring, and deep attenuation of ultrasound signal [19]. Patients must be diagnosed with early stage NAFLD simultaneously by two deputy director physicians.

Exclusion criteria: missing important clinical data; history of heavy alcohol consumption (≥30 g/day of ethanol or alcohol for men and ≥20 g/day for women in the past 12 months); cirrhosis; other causes of hepatic fat accumulation, including medications using (methotrexate, tamoxifen, and valproate), specific diseases (autoimmune hepatitis, total parenteral nutrition, congenital lipodystrophy, and Wilson disease); severe trauma or infections; thyroid disorder; severe heart disease or diabetes; pregnant women or breastfeeding women. Inclusion criteria for healthy controls were referenced to our previous studies [17].

### Serum sample collection and preservation

The standard protocol for sample collection and storage was formulated and followed based on our previous study [20] to control for possible sampling interference. Fasting blood collection was scheduled from 7:00 a.m. to 9:00 a.m. Fasting for at least 8 h, but no more than 16 h, was required [21]. Four milliliters of blood were collected from the vein of each enrolled subject and placed in a blood collection tube with an inert separator and coagulant; the tube was inverted 5–6 times and let stand upright. The blood samples were centrifuged (3000 rpm, 10 min), and then the supernatant was carefully collected. All samples were immediately stored at −80°C until metabolomics detection being performed.

### Sample pretreatment and metabolite extraction

After the samples were slowly thawed at 4°C in a salt-ice bath, 100 µl serum sample was precisely transferred by a pipettor to the 1.5 ml centrifuge tubes. Metabolites were extracted using a 400 µl methanol: water (4:1, v/v) solution with 0.02 mg/ml L-2-chlorophenylalanin as an internal standard, vortexed for 30 s, and extracted with low-temperature ultrasound for 30 min (5°C, 40 kHz). After extraction, the samples were left to stand at −20°C for 30 min. The sample was centrifuged (13000 rpm, 4°C, 15 min), and the supernatant was transferred to an UPLC-MS/MS injection vial for detection. Liquid chromatographic separations were performed on a Thermo Ultimate 3000 system (Thermo Fisher Scientific Inc., Waltham, MA, U.S.A.) equipped with a Waters ACQUITY UPLC® HSS T3 column (150 × 2.1 mm, 1.8 μm). Mass spectrometry operations were performed on a Thermo Q Exactive Focus mass spectrometer (Thermo Fisher Scientific Inc., Waltham, MA, U.S.A.) with spray voltages of 3.8 kV in positive ion mode (ESI+) and −2.5 kV in negative ion mode (ESI−), respectively.

A pooled quality control (QC) sample was prepared by mixing equal volumes of all samples. Metabolomics data have been deposited to the EMBL-EBI MetaboLights database (DOI: 10.1093/nar/gkz1019, PMID:31691833) with the identifier MTBLS4245. The complete dataset can be accessed here https://www.ebi.ac.uk/metabolights/MTBLS4245.

### Data preprocessing and annotation

A series of preprocessing of the raw data is required before analyses. Raw data were imported into the metabolomics processing software Progenesis QI (Waters Corporation, Milford, U.S.A.) for baseline filtering, peak identification, integration, retention time correction, and peak alignment, resulting in a data matrix of retention time, mass-to-charge ratio, and peak intensity. Mass spectra of these metabolic features were identified by using accurate mass data.

### Differential analysis and screening of differential metabolites

Multivariate statistical analysis was performed using ropls (Version 1.6.2, http://bioconductor.org/packages/release/bioc/html/ropls.html) of the R package from Bioconductor. The unsupervised principal component analysis (PCA), the partial least squares discriminate analysis (PLS-DA), and the orthogonal partial least squares discriminate analysis (OPLS-DA) were used to assess the overall distribution and global metabolic changes between comparable groups. The general clustering, trends, or outliers were visualized. A 200-permutation test was performed to test model reliability. Variable importance in the projection (VIP) was calculated in the OPLS-DA model. *P* values were estimated with Student’s *t-*test in single-dimensional statistical analysis. Metabolites with both VIP values greater than one and *P* values less than 0.05 were screened as differential metabolites. Volcano plot analysis and Student’s *t*-test were used to identify metabolites that were significantly affected in the dataset.

### Metabolic pathway analysis and bioinformatics analysis

After screening differential metabolites between the two groups, metabolic pathway analysis was performed to study the biological correlations using MetaboAnalyst V4.0 [22] (https://www.metaboanalyst.ca/). Kyoto Encyclopedia of Gene and Genomes (KEGG), Human Metabolome Database (HMDB), and Lipid Metabolites and Pathways Strategy (LIPID MAPS) were databases used. After KEGG orthologous groups (KO) annotation and pathway annotation of the differential metabolites, the metabolic pathway map of the differential metabolites was obtained. We performed functional and pathway analyses of the differential metabolites and classified them hierarchically. Hierarchical classification and pathway enrichment analysis was performed according to the pathway or function in which the metabolite was involved. The importance measure of each biomolecule was given a weighted score based on its relative importance using KEGG pathway topology analysis.

### Metabolomics detection and data analysis

Untargeted metabolomics testing based on UPLC-MS/MS technology was performed on all serum samples through the high-resolution mass spectrometer of Orbitrap Elite (Thermo-Finnigan). The metabolomics processing software Progenesis QI (Waters Corporation, Milford, U.S.A.) was used to data processing. Details on untargeted metabolomic detection and associated data assays are presented in Supplementary Data.

### Identification of the metabolite biomarkers and the construction of a diagnostic model

Discovery metabolite profiles and independent diagnostic metabolite profiles were obtained from the discovery set and the independent validation set, respectively. We then selected biomarkers from the serum metabolite group for further analysis and constructed a diagnostic model for the disease using 5-fold cross-validation of the random forest model. We further judged diagnostic effectiveness by evaluating the receiver operating characteristic curve and the probability of disease (POD) index. In addition, we performed validation in the independent validation set. This process referred to the methodology of our previous study [20]. ROC curve plotting was to assess the disease diagnostic ability of the model [22].

### Statistical analysis

Between groups comparison, independent sample *t*-tests were performed for normal continuous data, which were presented with mean ± SD; Mann–Whitney *U*-test were performed for nonnormal continuous data, which were presented as median (25–75 percentile). And χ2 test or Fisher’s exact test was performed for categorical variables. Categorical variables were presented with percentages. Significance was set at *P* values <0.05. The statistical analyses were performed using SPSS V.20.0 for Windows (SPSS, Chicago, Illinois, U.S.A.).

## Results

### Study design and demographics of the participants

**Figure 1:**
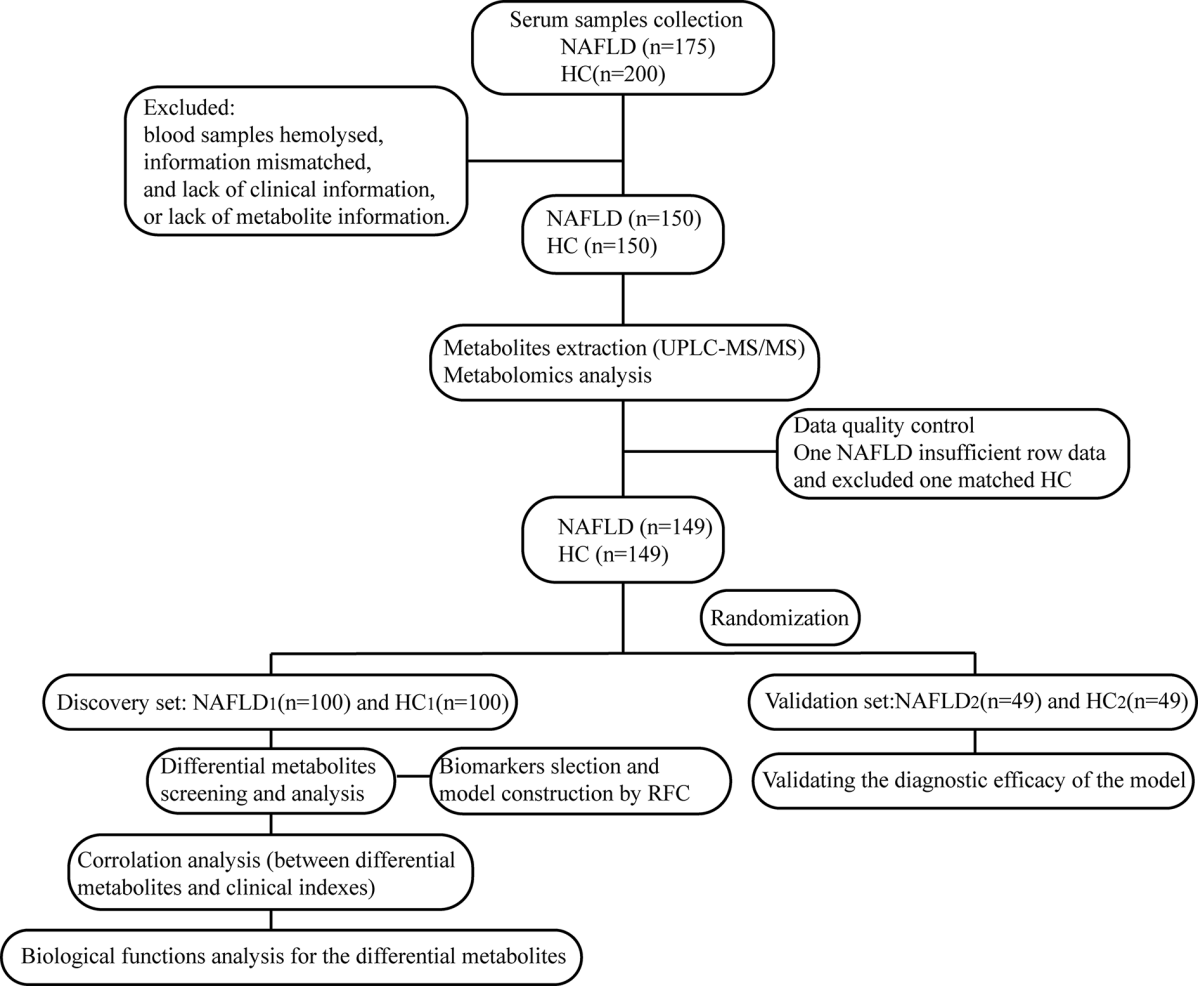
Study design and flow diagram After a strict selection and exclusion process, a total of 298 serum samples were included, including patients with early-stage NAFLD (*n*=149) and healthy controls (HC) (*n*=149). Two-thirds of the two groups were randomly selected as the training set (NAFLD_1_, *n*=100 vs HC_1_, *n*=100), and the remaining one-third as the validation set (NAFLD_2_, *n*=49 vs HC_2_, *n*=49). Serum samples were detected by UPLC-MS/MS. In the discovery set, we characterized serum metabolites profile and defined candidate biomarkers. Then, we explored biological function of the differential metabolites and constructed a diagnostic model for early-stage NAFLD using a random forest model; HC, healthy controls; NAFLD, nonalcoholic fatty liver disease patients; UPLC-MS/MS, ultra-performance liquid chromatography-mass spectrometry.

The workflow of the study was presented in Figure 1. We prospectively collected 375 serum samples. After strict inclusion and exclusion criteria, 298 samples were used for further analysis, 149 from patients with NAFLD, and 115 from healthy volunteers. In the present study, we characterized the serum metabolomics of patients with NAFLD and the healthy populations. Then, we identified key metabolites and explored biological function of the differential metabolites between NAFLD patients and healthy controls. Finally, we constructed a metabolite-based diagnostic model to distinguish NAFLD patients from healthy individuals.

The clinical characteristics of the two groups were shown in Supplementary Table S1. The differences in gender and age between the NAFLD and healthy controls (HC) groups were not statistically significant (*P*>0.05). Liver function indices such as alanine aminotransferase (ALT) and aspartate aminotransferase (AST) were higher in the early NAFLD group than that in the HC group, and differences had significant difference (*P*<0.05). Compared with the HC group, serum lipid indices, including triglycerides and total cholesterol were increased in the early NAFLD group (*P*<0.05). In addition, body mass index (BMI) and waistline in the early NAFLD patients were significantly higher (*P*<0.05).

### Differences in serum metabolite composition between the two groups

**Figure 2:**
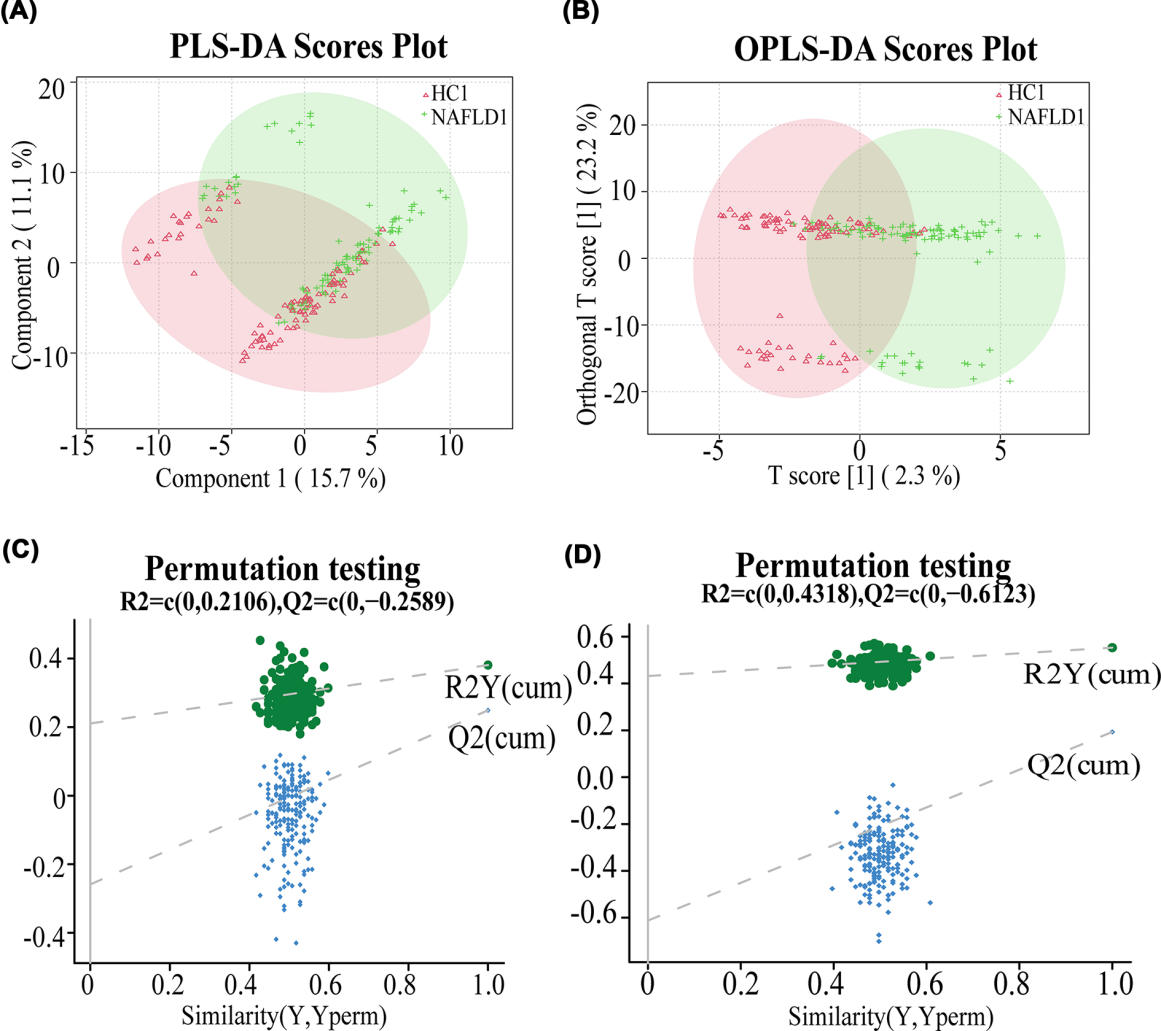
The composition of serum metabolites was different between the two groups The PLS-DA (**A**) and OPLS-DA (**B**) scores plot of NAFLD1 (green) and HC1 (red) groups. (**C**) The permutation test plot validated the reliability of the PLS-DA model. (**D**) The permutation test plot showed the OPLS-DA model was valid and not overfit; HC, healthy controls; NAFLD, nonalcoholic fatty liver disease patients; OPLS-DA, orthogonal partial least square discriminant analysis; PLS-DA, partial least squares discrimination analysis.

After importing the raw data (Supplementary Tables S2 and 3) into the metabolomics processing software Progenesis QI for data preprocessing and annotation, a total of 1090 metabolites were obtained for further statistical analysis (Supplementary Tables S4 and 5). In the discovery set, both the PLS-DA scores plot (Figure 2A) and the OPLS-DA scores plot (Figure 2B) showed separation of the two groups in the component one. The permutation test further validated the reliability and validity of the PLS-DA (Figure 2C) and the OPLS-DA model (Figure 2D), and there was no phenomenon of overfitting. The results showed significant difference in serum metabolite composition between the two groups. In addition, through the one-way analysis of variance combined with multivariate analysis, we identified the differential metabolites between groups (both VIP > 1 and *P* value < 0.05) which might serve as biomarkers for the diagnosis of early NAFLD patients. A total of 156 differential metabolites were screened out among the 1090 metabolites identified in the discovery set (Supplementary Table S6).

### The expression analysis of differential metabolites

Venn diagram revealed the overlap of differential metabolites (Supplementary Figure S1A). The volcano plots (Supplementary Figure S1B) displayed the overall profile of differential metabolites expression. Finally, we found that, compared with the HC group, the serum levels of 88 metabolites were significantly up-regulated while 68 metabolites were down-regulated in the NAFLD group (Supplementary Table S7). Among them, five metabolites, including 1-naphthylmethanol, rosavin, theophylline, phosphatidyl choline (PC) (14:1(9Z)/16:1(9Z)), and phosphatidyl serine (PS) (16:0/18:0) were significantly up-regulated in the serum of patients with early NAFLD. The significantly down-regulated metabolites were PC(P-19:1(12Z)/0:0), 2-hydroxyphenylacetic acid and lysophosphatidylcholine (LysoPC) (24:1(15Z)). In addition, according to the structure and function of metabolites, we performed the KEGG compounds classification to categorized and analyzed the differential metabolite. The results of the classification statistics, expressed as the differential metabolite classification bar chart (Supplementary Figure S1C) showed that of all the classifications of differential metabolites, lipids had the largest variety of compounds, including phospholipids, glycolipids, and fatty acids (Supplementary Table S8). In addition, the differential metabolites were also mainly classified into steroids, peptides (amino acids), carbohydrates, and carbohydrates.

### The pathway and function analysis for differential metabolites

The KEGG metabolic pathway hierarchical classification analysis (Supplementary Figure S2A and Supplementary Tables S9,10) showed a total of 116 differential metabolites were involved in 32 KEGG pathways in humans. According to KEGG classifications, the 32 individual KEGG pathways were classified into six categories, including organismal systems, metabolism, human diseases, genetic information processing, environmental information processing, and cellular processes. In the metabolism category, 57 differential metabolites were involved in 9 individual KEGG pathways, mainly including lipid metabolism, amino acid metabolism, and energy metabolism. Among them, lipid metabolism had 21 metabolites annotated. Subsequently, to further uncover the level of activity of the relevant biological pathways in the measured samples, we obtained a statistical histogram of the top 20 pathways containing the most differential metabolites (Supplementary Figure S2B). The histogram indicated that the largest variety of differential metabolites involved in glycerophospholipid metabolism and choline metabolism, demonstrating that these two biological pathways were relatively active.

KEGG pathway enrichment analysis (Supplementary Figure S2C) described the significantly enriched KEGG pathways (*P*<0.05), including caffeine metabolism, choline metabolism in cancer, retrograde endocannabinoid signaling, and sphingolipid metabolism (Supplementary Table S11). The KEGG topology bubble graph (Supplementary Figure S3A) showed five pathways with the highest impact to disease, including D-glutamine and D-glutamate metabolism, retinol metabolism, caffeine metabolism, alanine, aspartate and glutamate metabolism, and the ether lipid metabolism. Metabolic pathway network map showed in Supplementary Figure S3B–F and the Supplementary Table S12 demonstrated the specific locations and roles of metabolites in these five metabolic pathways, respectively.

### Potential diagnostic value of serum metabolites in the early NAFLD

**Figure 3:**
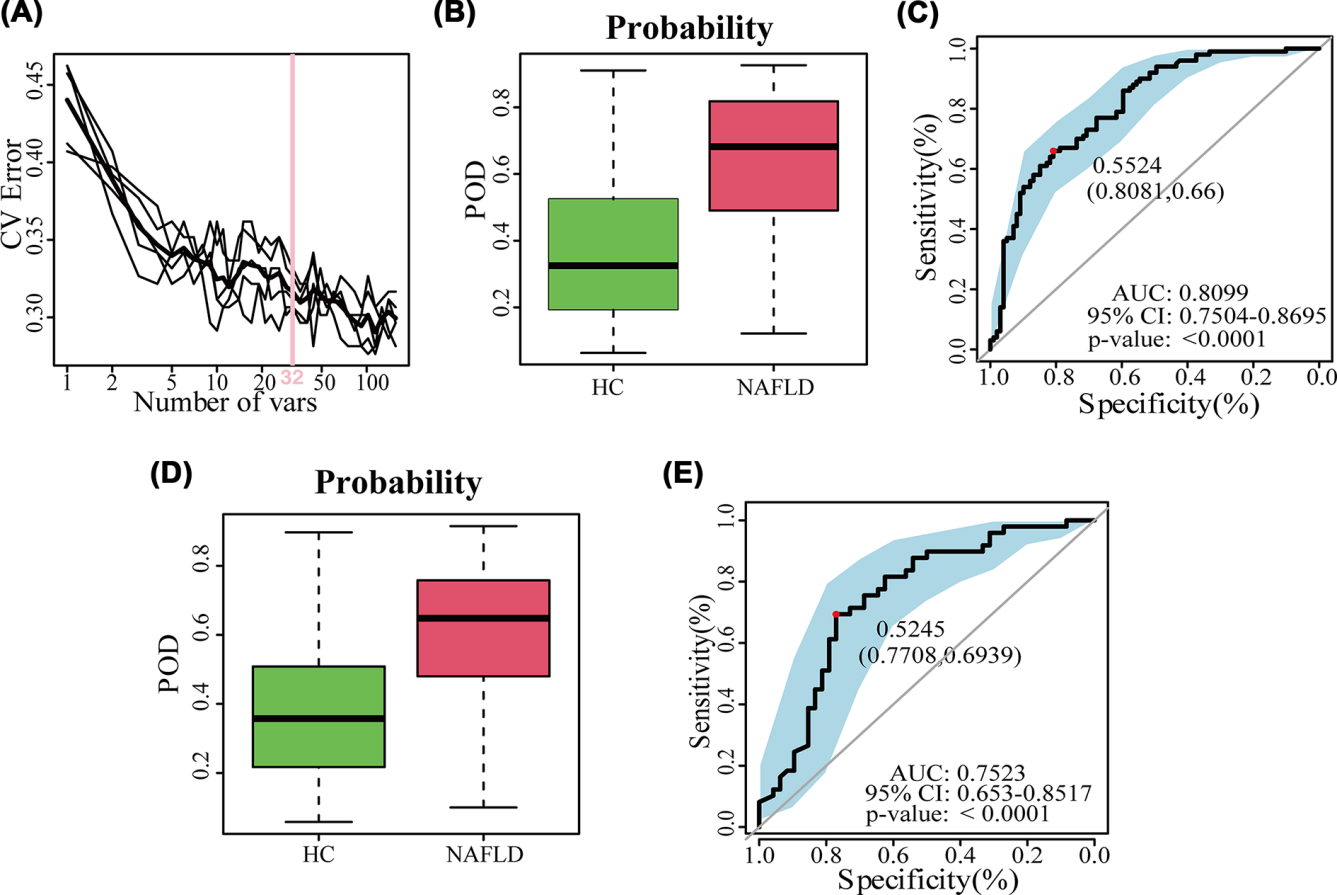
The identification of serum metabolite markers and construction of diagnostic model for early NAFLD (**A**) The cross-validation curves showed 32 serum metabolites were selected as the optimal markers. (**B,D**) The POD index was significantly higher in NAFLD than HC groups both in the discovery set (B) and in the validation set (D). (**C**) The receiver operating characteristic curve in the discovery set. (**E**) The receiver operating characteristic curve in the validation set; AUC, area under the curve; CV error, cross-validation error; HC, healthy controls; NAFLD, nonalcoholic fatty liver disease patients; POD, probability of disease.

To elucidate the diagnostic value of the serum metabolome for early NAFLD, a random forest prediction model was constructed that could distinguish the patients with early NAFLD and healthy populations. Initially, we constructed a random forest classifier model in the discovery set (NAFLD_1_, *n*=100 vs HC_1_, *n*=100). Based on the random forest model and a 5-fold cross-validation, we finally selected 32 serum metabolites as the best biomarkers, as shown in the cross-validation curves (Figure 3A). The importance distribution map (Supplementary Figure S4) illustrated the diagnostic importance of the 32 serum metabolite markers in the model. And the POD index was significantly higher in the early NAFLD patients than in the HCs (Figure 3B), with an area under the curve (AUC) of 80.99% (95% CI: 75.04–86.95%, *P*<0.0001) (Figure 3C). These results demonstrated that the diagnostic potential of this classifier for early NAFLD. Moreover, in the validation set (NAFLD_2_, *n*=49 vs HC_2_, *n*=49), the POD index was significantly increased in the NAFLD group compared with healthy individuals (Figure 3D), and the POD index reached an AUC value of 75.23% between the NAFLD and HC groups with the 95% CI of 65.3–85.17%, *P*<0.0001 (Figure 3E). These results suggested that the model for early stage NAFLD had powerful diagnostic efficacy.

### Correlation between serum metabolites and NAFLD disease status

**Figure 4:**
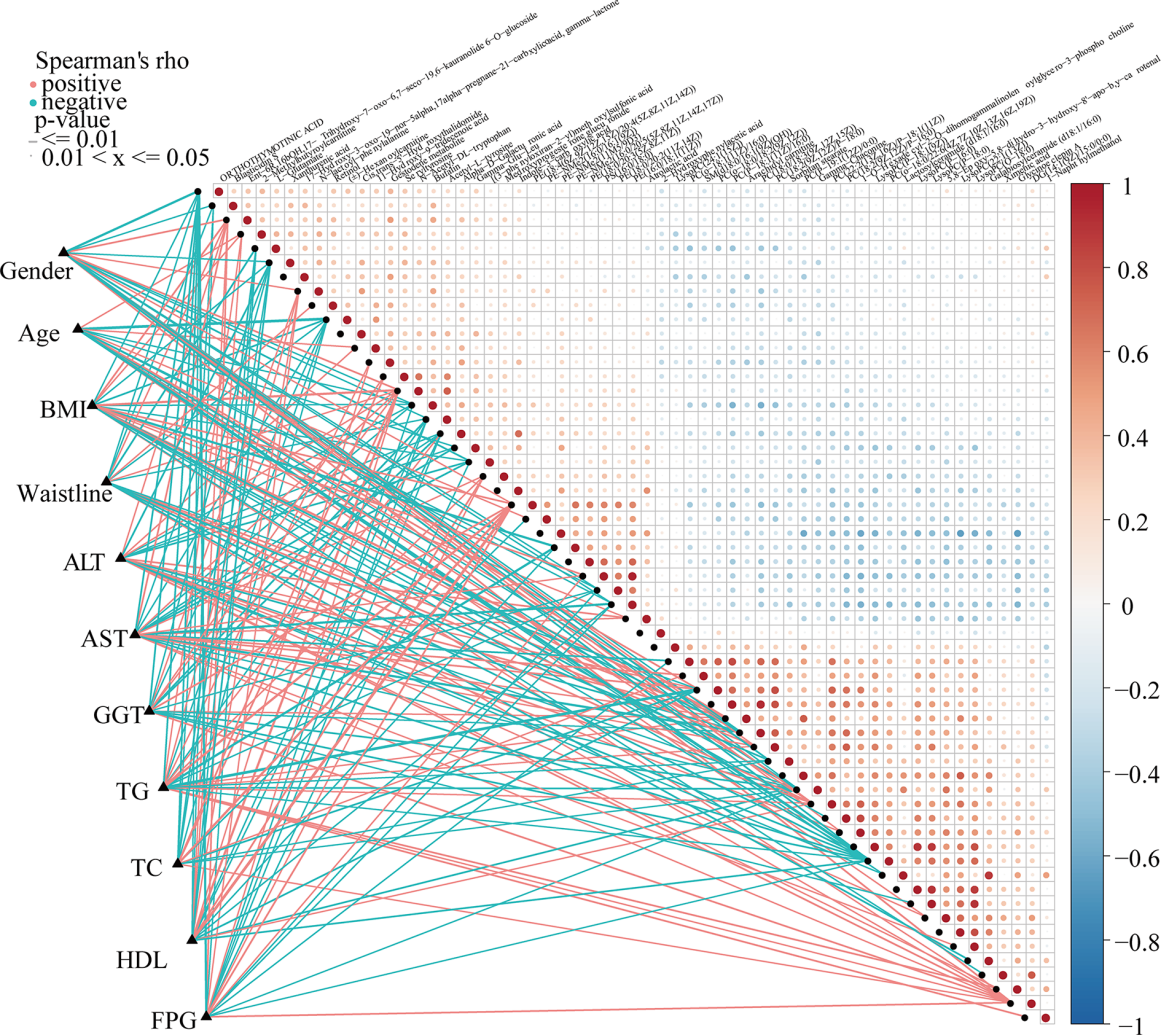
Correlation between serum metabolites and the clinical indicators of early NAFLD The distance correlation matrix plots displaying the partial Spearman’s correlation among the 59 serum differential metabolites and 11 clinical indicators of early NAFLD patients. Positive values (red) indicate positive correlations. Negative values (blue) indicate inverse correlations. Solid lines represent that *P*≤0.01. Dotted lines represent that 0.01<*P*≤0.05. Intensity of shading in circles is proportional to the magnitude of the association; ALT, alanine aminotransferase; AST, aspartate aminotransferase; BMI, body mass index; FPG, fasting plasma glucose; GGT, γ-glutamyl transferase; HDL, high-density lipoprotein; NAFLD, nonalcoholic fatty liver disease patients; TC, total cholesterol; TG, triglycerides.

To explore the potential correlation between clinical indicators and differential metabolites, Spearman’s rank test correlation analysis was performed in the present study. The distance correlation matrix plots (Figure 4) showed correlations existed between the 59 serum differential metabolites and important clinical indicators of early NAFLD patients, such as body mass index, serum lipids, and liver function in patients with early NAFLD and healthy populations. We found that BMI, ALT, AST, waist circumference, and triglycerides were positively correlated with 25 serum metabolites. And the gender was positively associated with 26 serum metabolites. The results showed that BMI was positively correlated with the serum levels of 26 differential metabolites, including N-lactoyl-phenylalanine (*P*<0.05, rho = 0.351), 2-methylbutyroylcarnitine (*P*<0.001, rho = 0.378), L-glutamate (*P*<0.001, rho = 0.453), 11-hydroxy-9-tridecenoic acid (*P*<0.05, rho = 0.257), and medroxyprogesterone glucuronide (*P*<0.001, rho = 0.288). However, metabolites, such as PC(P-19:1(12Z)/0:0) (*P*<0.05, rho = −0.473), and γ-chaconine (*P*<0.05, rho = −0.465), were negatively correlated with waist circumference, glutamyl transpeptidase, and serum levels of triglycerides. And 1-naphthalenemethanol did not correlate with any clinical indicators.

## Discussion

As a metabolic-associated fatty liver disease, NAFLD has become the most common chronic liver disease worldwide, bringing a huge burden on both the individual and the public health care system globally [5]. Previous studies [10,16] have demonstrated the metabolomic changes in patients with NAFLD and the diagnostic value, which indicates that metabolomic analysis for patients with early NAFLD is a promising approach that can identify novel biomarkers as targets for the diagnosis of NAFLD. Hence, the present study reported the serum metabolomic profiles of early NAFLD patients (*n*=149) and healthy volunteers (*n*=149) from central China by UPLC-MS/MS and the bioinformatics analysis technology.

Our study revealed a significant difference in serum metabolites composition between patients with early NAFLD and healthy individuals. In the discovery set, we identified 156 differential metabolites. Among them, 88 metabolites were up-regulated in the NAFLD patients. The classification results showed that the differential metabolites were mainly classified into lipids (fatty acids, phospholipids, and glycolipids), steroids, and amino acids. Importantly, amino acids are clear difference in serum metabolites in NAFLD compared with the HC group and amino acid metabolism was shown to be associated with NAFLD [12,23,24], which is consistent with the results in the present study. In addition, plasma isoleucine levels were significantly increased, while valine and asparagine levels were significantly decreased in patients with NAFLD [12,16]. Previous studies have reported that kynurenic and kynurenine acid resulting from tryptophan metabolism are associated with immune cell activation and systemic inflammation [23]. In addition, isoleucine, as a kind of branched-chain amino acid (BCAA), was involved in gluconeogenesis and insulin resistance [14,16]. Moreover, animal serum metabolomics studies revealed a significant increase in serum saturated fatty acid levels in mice with NAFLD induced by the high-fat diet (HFD) [25]. In NAFLD, the altered metabolic pathways are associated with abnormal bile acid metabolism, oxidative stress, and inflammation, and inflammatory infiltration of the liver plays an essential role in the pathogenesis of NAFLD [25]. However, the impact of such changes on the prevalence of NAFLD needs to be further investigated.

More surprisingly, Spearman’s correlation analysis showed that clinical indicators were correlated with differential metabolites in the present study. We observed positive correlations between sex and 26 serum metabolites. Li et al. [26] reported that gender, triglycerides, glucose, and BMI were significantly positively associated with the incidence of nonobesity NAFLD. Gender may promote the development of NAFLD by affecting insulin resistance and glucolipid metabolism [26]. Hence, we considered that clinical indicators were associated with the incidence and severity of NAFLD. Moreover, we also found that triglycerides, glucose, and BMI were correlated negatively with LysoPC (24:0), and the serum level of LysoPC (24:0) was increased in patients with NAFLD. LysoPC can activate G protein-coupled receptor 119 and promote insulin release [27]. However, as a marker for some liver diseases, the serum level of LysoPC was reduced in chronic liver injury-related cirrhosis and hepatocellular carcinoma, and it was related to the mortality risk [28]. In addition, Krautbauer et al. [29] reported that the chemotactic effect of LysoPC on serum interleukin-8 and monocyte chemotactic protein-1 might generate a proinflammatory effect. Therefore, we hypothesize that the increased serum LysoPC levels in NAFLD patients may contribute to liver injury and NAFLD progression through pro-inflammatory effects.

Notably, after screening out the serum differential metabolic markers, we further explored their biofunction and relevance to early NAFLD through pathway analysis. Lipopolysaccharide can cause metabolic endotoxemia, producing insulin resistance and promoting fatty liver progression [30]. More importantly, studies have demonstrated that clustering differentiation 44 significantly increases macrophage activation via saturated fatty acids and lipopolysaccharides. Moreover, clustering differentiation 44 enhances hepatic steatosis by regulating hepatic macrophage polarization and infiltration, leading to the progression of NAFL to NASH [31]. In our present study, KEGG enrichment analysis results showed statistically significant (*P*<0.05) differences in phenylalanine metabolism between the NAFLD and HC groups. The only differential metabolite involved in the phenylalanine metabolism pathway was down-regulated 2-hydroxyphenyl acetic acid. Animal and cellular studies proved that phenylacetic acid produced by phenylalanine catabolism promoted steatosis and may increase hepatic lipid accumulation by increasing branched-chain amino acid utilization in the tricarboxylic acid cycle [32]. The results suggested that metabolite pathways influenced by the differential metabolites between the two groups might influence the progression of NAFLD.

Many previous studies have reported the potential and value of metabolomics-based serum metabolite markers in the diagnosis and staging prediction of NAFLD. Recently, Satish et al. [12] reported that using random forest analysis and recursive partitioning could distinguish healthy individuals from NAFLD patients with an error rate of 8%. Masarone et al. [16] also demonstrated that untargeted metabolomics could be used to diagnose and assess different stages of NAFLD. In our present study, we identified 32 optimal serum biomarkers for NAFLD. The NAFLD classifier exhibited strong diagnostic potential (AUC = 0.8099) in the discovery set and was successfully validated in the validation set. Compared with Masarone et al., our study used multiple biomarkers to build the diagnostic model, which increased the stability and reliability of the model. Moreover, we also used the validation set to verify the performance of this model and achieved good results, which further illustrated the feasibility of this model. Hence, the model could distinguish early NAFLD patients from healthy individuals, suggesting that serum metabolic markers had great potential as a new complementary diagnostic tool for early NAFLD.

Our study has some limitations. The liver function indices in patients with early-stage NAFLD are within the normal range, and early clinical symptoms are not obvious. Many patients are not able to aware of the long-term risks of the disease. Most patients were diagnosed with fatty liver mainly by liver ultrasound during the routine physical examination and did not agree to a liver biopsy for a definitive diagnosis. Our inability to perform biopsies resulted in the lack of longitudinal samples. However, we set strict inclusion criteria. Hence, our results are more practical for clinicians. Finally, there can be heterogeneity in the metabolite composition of serum samples with different population characteristics. Therefore, further integrating targeted and untargeted metabolomic analyses, multicenter, large-scale metabolomic studies are needed to validate the biological functions of differential metabolites and assess the diagnostic efficacy of the models. Further studies are needed to translate these findings for their better application in clinical practice in the future.

In conclusion, the present study reported the compositional and functional alterations in early NAFLD associated serum metabolites, identified specific serum metabolite markers. Importantly, based on the key serum metabolite markers, this study also established a diagnostic model for early NAFLD and achieved good diagnostic efficacy. With the further development of metabolomics technologies, the application of metabolomics in the diagnosis and prognosis of early-stage NAFLD might have great potential and promise in the future.

## Resume

**Xuemei Wang, Benchen Rao** and **Haiyu Wang** are the Ph.D. candidate in Internal Medicine (Liver Disease) at Zhengzhou University, engaged in the mechanism research of the interaction between gut microecology and serum metabolomics on nonalcoholic fatty liver disease.

**Chao Liu** is a professional experimenter of Shanghai Mobio Biomedical Technology Co., Ltd., Shanghai, China.

**Zhigang Ren**, Ph.D., associate professor, hepatologist at the First Affiliated Hospital of Zhengzhou University, engaged in innovative diagnosis and treatment of liver disease and clinical and basic research on human microecology, young editorial board member of Military Medica Research.

**Zujiang Yu**, Ph.D., Professor, Chief Physician of the Department of Hepatology, The First Affiliated Hospital of Zhengzhou University. Established the first noninvasive diagnostic marker for early-stage liver cancer based on gut microbiota.

## Supplementary Material

Supplementary Figures S1-S4

Supplementary Tables S1-S12

Supplementary Data

## Data Availability

Metabolomics data have been deposited to the EMBL-EBI MetaboLights database (DOI: 10.1093/nar/gkz1019, PMID:31691833) with the identifier MTBLS4245. The complete dataset can be accessed here https://www.ebi.ac.uk/metabolights/MTBLS4245. All data can be obtained by contacting the corresponding author.

## Abbreviations

ALT: alanine aminotransferase
AST: aspartate aminotransferase
AUC: area under the curve
BCAA: branched-chain amino acid
HC: healthy controls
HFD: high-fat diet
NAFLD: nonalcoholic fatty liver disease
VIP: variable importance in the projection
