# Supplementary material for: Serum metabolome alterations in patients with early nonalcoholic fatty liver disease
Source: Biosci Rep. 2022 Oct 18;42(10):BSR20220319. doi: 10.1042/BSR20220319 (PMC9583763; doi:10.1042/BSR20220319)
Supplement: Supplementary Figures S1-S4 [file BSR-2022-0319_supp.pdf]

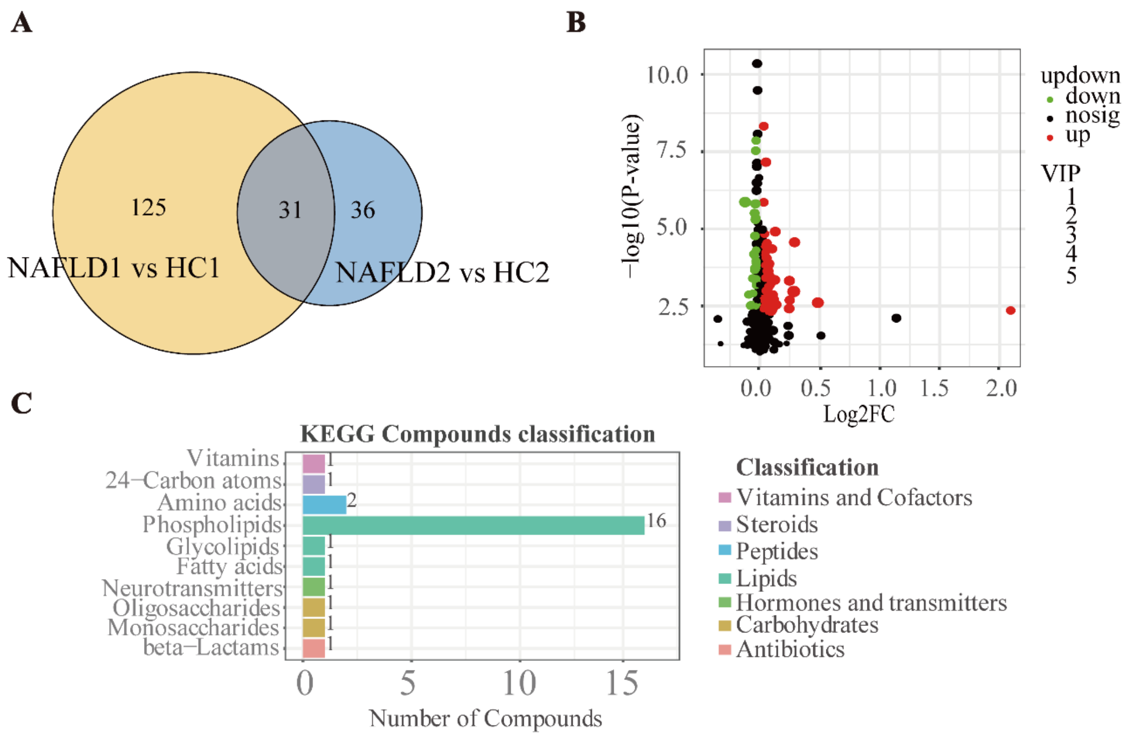

Supplementary Figure 1. Bioinformatics analysis of differential metabolite profiles.

(A) Venn diagram of differential metabolites. 31 of the differential metabolites were shared between the training set (yellow) and the validation set (blue). (B) Volcano plot for the differential metabolites. The horizontal axis (log2FC) is the value of the fold change in metabolite expression difference between the two groups, and the vertical axis (-log10(p-value)) is the value of the statistical test for the difference in metabolite expression change, with higher values indicating more significant expression differences. Each point in the graph represents a specific metabolite, and the size of the point indicates the VIP value. The red and green dots indicate significantly upregulate and downregulated metabolites, respectively. (C) Histograms of differential metabolites. The horizontal axis represents the number of metabolites. The vertical axis represents the metabolite classification. FC, fold change; VIP, variable important in the projection.

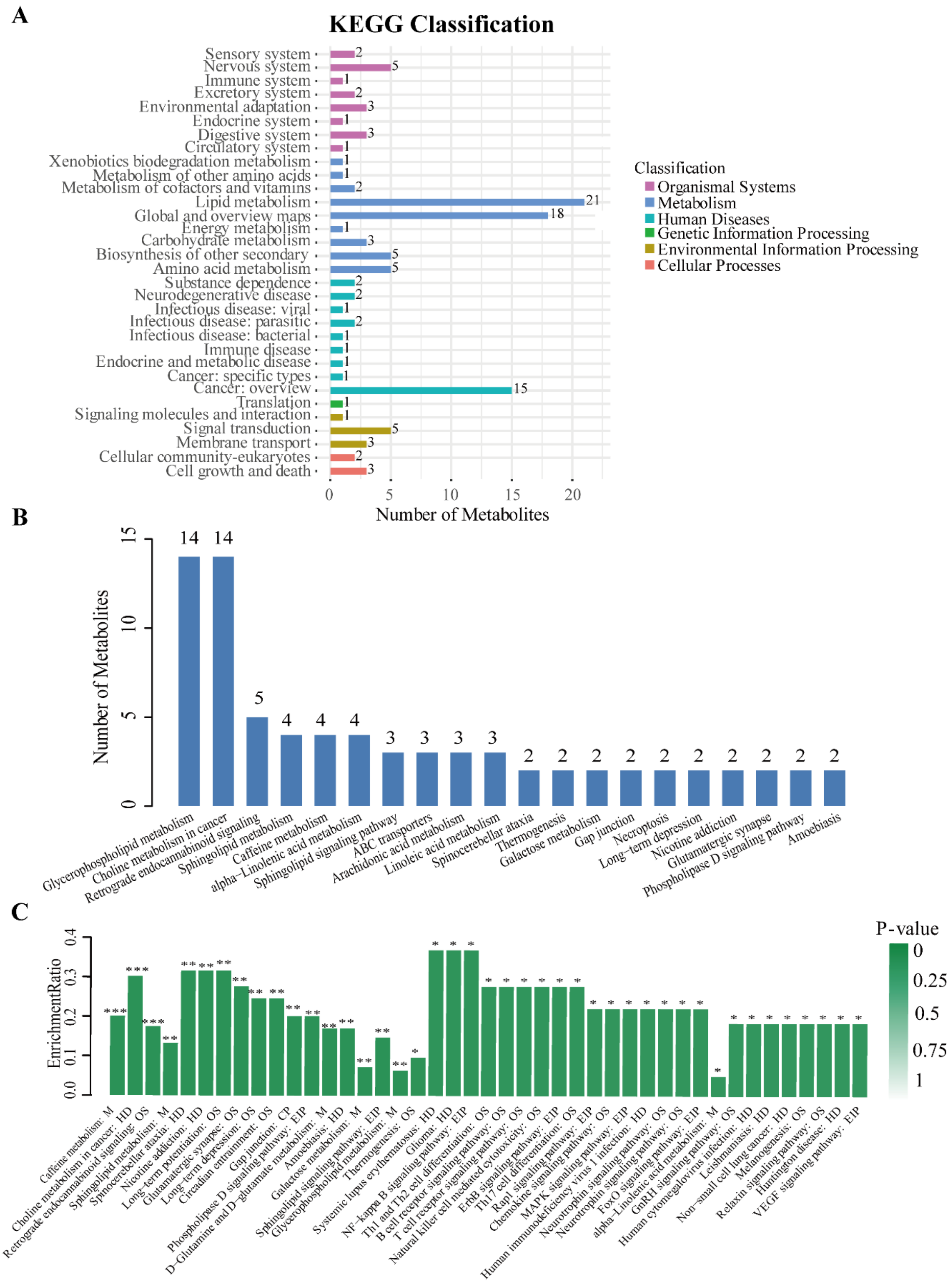

Supplementary Figure 2. The pathway and function analysis for the differential metabolites.

(A) The hierarchical classification analysis of KEGG metabolic pathway. The ordinate is the name of the KEGG metabolic pathway, and the abscissa is the number of metabolisms annotated to this

pathway. Pathways with different colors indicate that they belong to different categories. According to the KEGG hierarchical database, the KEGG database is divided into 4 levels (brite), which are represented by A, B, C and D respectively. According to KEGG classifications, the Brite A includes 6 categories, corresponding to different colors in the legend on the right of the figure; brite B belongs to a small branch in brite A, which corresponds to the ordinate in the figure; brite C is a specific metabolic pathway; brite D is the gene product in the pathway (metabolite). (B) Statistical histogram of the top 20 pathways containing the largest number of differential metabolites. The higher the column is, the more active the biological pathway is in the measured samples. Therefore, the higher the order of the pathway can be selected for further analysis according to different research purposes. (C) Histogram of KEGG pathway enrichment analysis for each group of differential metabolites. Each column in the figure is a channel. The abscissa text indicates the name and classification of the pathway. The color indicates the significance of the enrichment, that is, the P value, and the darker the color, the more significantly enriched the pathway is. The color gradient on the right represents the magnitude of the p-value. *** $P<0.001$; ** $P<0.01$; * $P<0.05$.

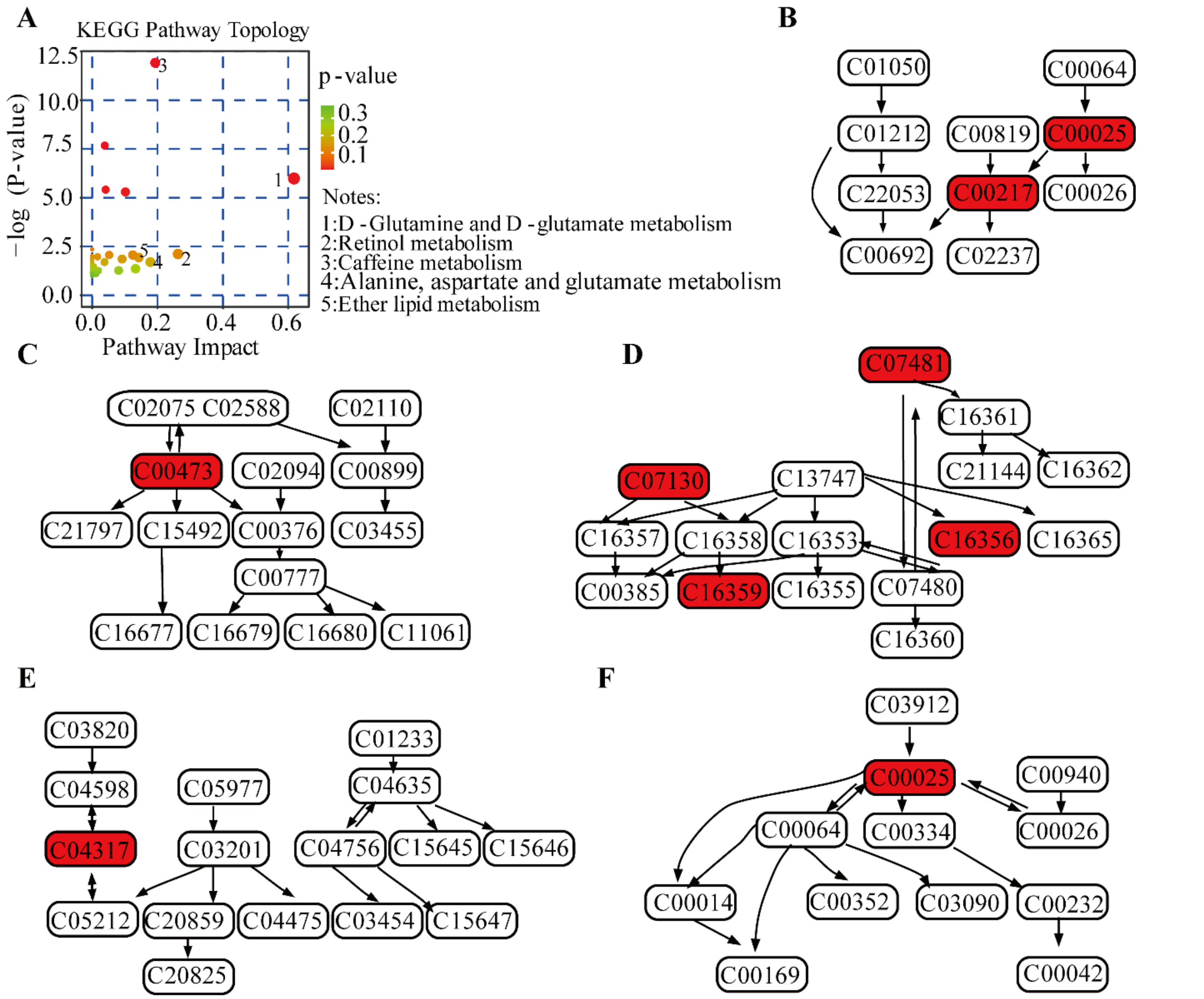

Supplementary Figure 3. **Topological analysis of metabolic pathway of differential metabolites.**

(A) KEGG topology bubble graph of metabolic pathway. Each bubble represents a KEGG pathway. The horizontal axis indicates the relative importance of metabolites in the pathway. The vertical axis indicates the enrichment significance of the pathway the metabolites involvement in, -log2(p-value). The larger the bubble, the higher the importance value. Bubble color indicates the p-value of pathway enrichment. (B to F) The KEGG metabolic pathway network diagram. Labels within the boxes corresponded to their KEGG identifiers for metabolites, or the KEGG compound ID. Network diagram of metabolite reactions extracted from the reactions in the KEGG pathway. Each graph represents a KEGG pathway; metabolites in the white background indicate metabolites involved in this pathway; pathways in the red background indicate differential metabolites identified in this time. (B) D-glutamine and D-glutamate metabolism pathway, involving

metabolites C00025 (L-glutamate) and C00217 (L-glutamate). (C) Retinol metabolism, and the metabolite was C00473 (retinol). (D) Caffeine metabolism, and the metabolites were C07130 (theophylline), C16356 (1,7-dimethyluric acid), C16359 (1-methyluric acid) and C07481 (caffeine). (E) Lipid metabolism pathway, involving C04317 (LysoPC(O-18:0)). (F) Alanine, aspartate, and glutamate metabolism, C00025 (L-glutamate).

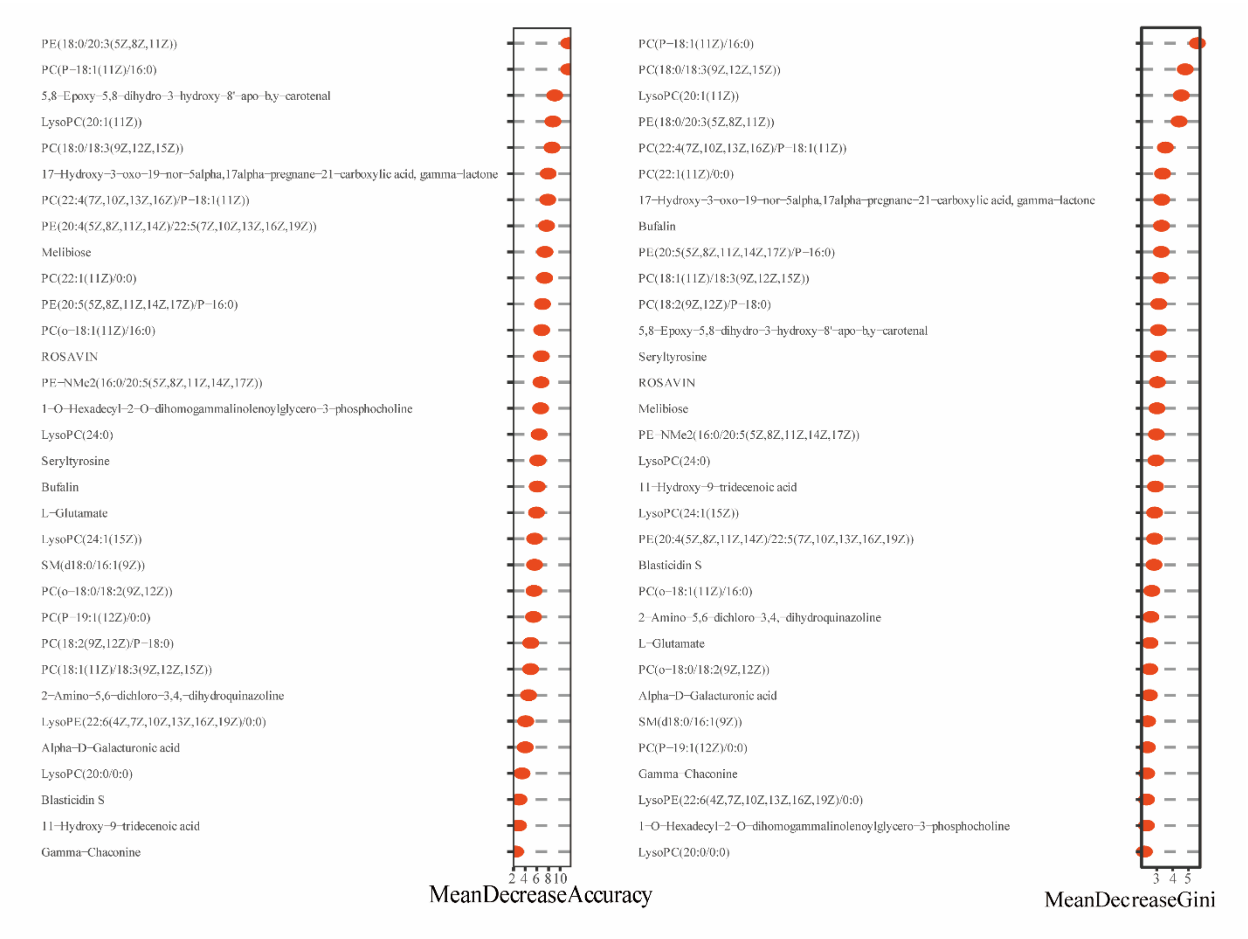

Supplementary Figure 4. The importance distribution map of the 32 serum metabolite markers in the model.

It illustrated the diagnostic importance of the 32 serum metabolite markers in the model.
