# Supplementary material for: Serum metabolome alterations in patients with early nonalcoholic fatty liver disease
Source: Biosci Rep. 2022 Oct 18;42(10):BSR20220319. doi: 10.1042/BSR20220319 (PMC9583763; doi:10.1042/BSR20220319)
Supplement: Supplementary Data [file BSR-2022-0319_supp2.pdf]

Supplementary materials

1. Consent informed

Informed consent form for scientific research

(Translated from Chinese)

Dear participants,

We are from Department of Infectious Diseases, the First Affiliated Hospital of Zhengzhou University. We will free of charge help you monitor your healthy condition and record your clinical information and healthy/disease status or disease progression process. The collected serum samples from participants in hospital will be used for scientific research. These results and data from the hospital electronic medical records will provide auxiliary data for clinical diagnosis and treatment, and that will be used for scientific research. Thank you for your corporation.

Number:	Diagnosis:

The information that we collect from this research project will be kept confidential. Information about you will be collected during the research will be put away and no-one but the researchers will be able to see it. Any information about you will have a number on it instead of your name. Only the researchers will know what your number is and we will lock that information up with a lock and key. It will not be shared with or given to anyone except our research team.

The knowledge that we get from doing this research will be shared with you through community meetings before it is made widely available to the public. Confidential information will not be shared. There will be small meetings in the community and that will be announced. After these meetings, we will publish the results in order that other interested people may learn from our research.

I have read the foregoing information, or it has been read to me. I have had the opportunity to ask questions about it and any questions that I have asked had been answered to my satisfaction. I consent voluntarily to participate as a participant in this research.

Print Name of Participant__________________

Signature of Participant __________________

Date ___________________________

Day/month/year

A literate witness must sign (if possible, this person should be selected by the participant and should have no connection to the research team). Participants who are illiterate should include their thumb prints as well.

I have witnessed the accurate reading of the consent form to the potential participant, and the individual has had the opportunity to ask questions. I confirm that the individual has given consent freely.

Print name of witness_____________________ AND

Thumb print of participant

Signature of witness ______________________

Date ________________________

Day/month/year

Statement by the researcher/person taking consent

I have accurately read out the information sheet to the potential participant, and to the best of my ability made sure that the participant understands that the following will be done:

1. We will free of charge help you monitor your healthy condition and record your clinical information and healthy/disease status or disease progression process.

2. These data from hospital electronic medical records will be used for scientific research.

3. The collected blood or serum samples will be used for scientific research.

I confirm that the participant was given an opportunity to ask questions about the study, and all the questions asked by the participant have been answered correctly and to the best of my ability. I confirm that the individual has not been coerced into giving consent, and the consent has been given freely and voluntarily.

A copy of this ICF has been provided to the participant.

Print Name of Researcher/person taking the consent________________________

Signature of Researcher /person taking the consent__________________________

Date ___________________________

Day/month/year
